# Anthelmintic Activity of Antioxidants: In Vitro Effects on the Liver Fluke *Opisthorchis felineus*

**DOI:** 10.3390/pathogens10030284

**Published:** 2021-03-02

**Authors:** Viatcheslav A. Mordvinov, Denis V. Ponomarev, Yuri V. Pakharukov, Maria Y. Pakharukova

**Affiliations:** 1 Institute of Cytology and Genetics, Siberian Branch of Russian Academy of Science, 10 Lavrentiev Ave., 630090 Novosibirsk, Russia; mordvin@bionet.nsc.ru (V.A.M.); ponomarevd@mail.ru (D.V.P.); 2 Department of Physics, Monitoring and Diagnostic Methods, Industrial University of Tyumen, 38 Volodarskogo Str., 625000 Tyumen, Russia; pacharukovyu@yandex.ru; 3 Department of Natural Sciences, Novosibirsk State University, 2 Pirogov Str., 630090 Novosibirsk, Russia

**Keywords:** antioxidant, flavonoid, liver flukes, praziquantel, coenzyme Q

## Abstract

Currently, molecular parasitologists are searching for new agents against trematodiases. Redox metabolism is important for parasites as far as long-lived adult parasites inside a mammalian host are exposed to redox challenges. Antioxidants have been poorly studied as anthelmintic agents, in particular against the foodborne trematodes. Study of in vitro anthelmintic activity of nonenzymatic natural and synthetic antioxidants of various chemical structures was performed using standard motility and mortality assays against juvenile and adult *Opisthorchis felineus* worms. Promising agents have been found among both natural and synthetic compounds. The mitochondria-targeted antioxidant SkQ1 [10-(6′-plastoquinonyl)decyltriphenylphosphonium] in motility assays was as effective (half-maximal inhibitory concentration [IC_50_] 0.6–1.4 μM) as praziquantel (IC_50_ 0.47–1.4 μM), and SkQ1 was significantly more effective than praziquantel in mortality assays. Moreover, extensive tegument damage of the adult fluke was revealed after SkQ1 treatment. Flavonoids manifested potency too, with IC_50_ values in a micromolar range (5.1–17.4 μM). Other natural and synthetic compounds tested against helminths were significantly less effective than praziquantel. Results of our study indicate that SkQ1 and flavonoids have high anthelmintic activities against the liver flukes. We propose that structure–activity relationship research might be worthwhile based on the structures of the most effective substances.

## 1. Introduction

Liver fluke infections caused by foodborne trematodes *Clonorchis sinensis (C. sinensis)*, *Opisthorchis viverrini (O. viverrini)* and *Opisthorchis felineus* (*O. felineus*) affect millions of people and have a major socioeconomic impact worldwide. These trematodiases have similar pathogenesis. Humans become infected by eating undercooked or raw Cyprinidae fish [1,2]. The infections usually last for years and are associated with hepatobiliary morbidities including cholangiocarcinoma [1,2,3,4]. *O. felineus* is endemic in Europe and Russia, *C. sinensis* in China and the Republic of Korea, and *O. viverrini* in Thailand and Lao PDR. Infections with *O. viverrini* and *C. sinensis* are regarded by the International Agency for Research on Cancer (IARC) as definite causes of cancer [1].

Over the past three decades, praziquantel (PZQ) has been a mainstay treatment of liver fluke infections. This drug is also used to treat most trematode infections, including schistosomiasis, and is often employed in mass drug administration programs for the control of neglected tropical fluke diseases in low- and middle-income countries [5]. Such widespread and prolonged use of PZQ may lead to the emergence of resistant variants of parasitic worms. Therefore, the discovery and development of novel efficacious drugs to control and treat helminthiases are some of the top research priorities [5]. 

Redox metabolism is important for parasites as far as long-lived adult parasites inside mammals are exposed to redox challenges including those possibly from their own parasitic aerobic metabolism [6,7]. Thus, parasite survival may depend on the ability to maintain the necessary balance between oxidation and antioxidation. The importance of redox metabolism for parasites is underscored by the number of relevant genes in helminth genomes, levels of expression of these genes, and the presence of relevant proteins in the secretome. The secretory product of helminths includes, among other substances, a large set of proteins involved in redox reactions, in particular, TPx, thioredoxin, glutathione peroxidase, glutathione transferase, and thioredoxin glutathione reductase. By mRNA abundance, some relevant genes in an adult worm are among the 10 most expressed genes [8]. 

Redox system inhibition by certain compounds can be an effective anthelmintic strategy. In particular, it is known that trypanosome alternative oxidase is a promising drug development target [7]. It is also known that cytochrome P450 (CYP) is important for helminth survival and is a pharmaceutical target in many parasitic organisms, including *Trypanosoma cruzi*, schistosomes, and *O. felineus*. CYP inhibitors have a pronounced anthelmintic activity at micromolar to nanomolar concentrations [9,10]. 

**Figure 1:**
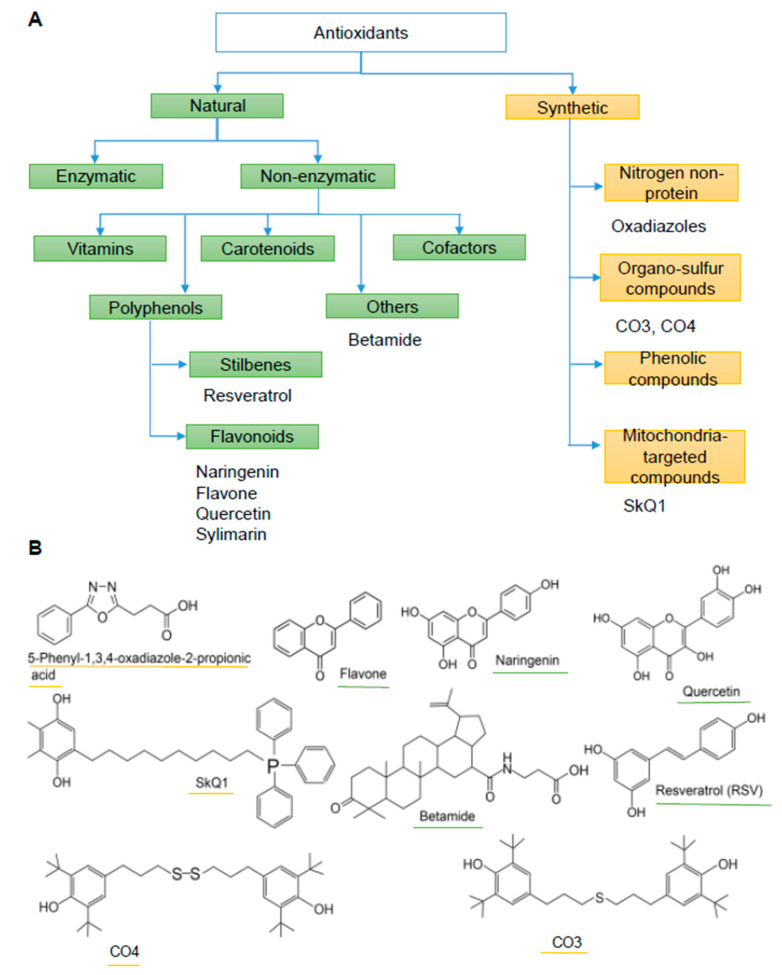
Schematic representation of antioxidant classes (**A**) and structures of the antioxidants (**B**). (**A**) Green words represent natural antioxidants, and yellow ones denote synthetic antioxidants.

Antioxidants as anthelmintic agents began to be studied recently and some of them show a good antiparasitic potential [6,11,12,13,14,15]. For instance, the oxadiazoles designed for the inhibition of thioredoxin glutathione reductase of helminths exert strong anthelmintic action against *T. cruzi*, *Leishmania* spp. [11], and *Schistosoma japonicum* [6]. Some of the known nonenzymatic natural antioxidants are minerals, vitamins, polyphenols, and terpene antioxidants (Figure 1). Polyphenols are a class of phytochemicals [12,13] that consists of phenolic acids, flavonoids, and stilbenes (Figure 1). Synthetic antioxidants are often a good substitute for natural ones and usually contain several functionally significant groups. Although there is evidence of the effectiveness of certain antioxidants against parasitic protozoa, antioxidants have been barely studied as anthelmintic agents, particularly against *O. felineus*.

Here, we studied the in vitro anthelmintic properties of antioxidant substances against adult and juvenile *O. felineus* liver flukes by the motility and mortality assays. We tested the anthelmintic activity of natural and synthetic antioxidants of various chemical structures (Figure 1), choosing them from different classes; in particular, we selected six natural compounds, of which four substances belong to the flavonoid group (quercetin, silymarin, naringenin, and flavone) as the base compounds for all flavonoids. In addition, we investigated a stilbene (resveratrol) and a triterpene antioxidant called betamide. Among the synthetic antioxidants, we selected two sulfur-containing organic substances (Figure 1), a nitrogen-containing organic nonprotein antioxidant from the oxadiazole class, and a mitochondria-targeted antioxidant: 10-(6′-plastoquinonyl)decyltriphenylphosphonium [also known as SkQ1].

## 2. Results

### 2.1. Motility Assays

**Figure 2:**
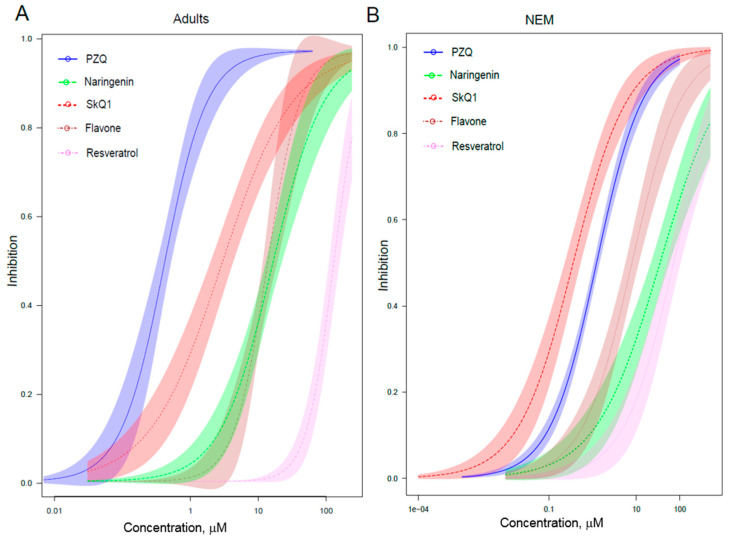
The concentration-dependent inhibitory activity of resveratrol, 10-(6′-plastoquinonyl)decyltriphenylphosphonium (SkQ1), praziquantel (PZQ), naringenin and flavone against the motility of the adult (**A**) and newly excysted metacercariae (NEM) (**B**) *O. felineus* liver flukes. Four-parameter logistic regression was used to calculate the half-maximal effective dose (IC_50_) and 95% confidence interval (‘drc’ R 3.6.0 package).

The current gold standard for measuring drug effectiveness for most helminth parasites is in vitro testing performed by microscopy, which included parasite counting and evaluation of motility and morphology [6,9,10,15]. Although reduced motility is not an ideal metric of mortality, it is a reasonable reduced viability analogue when screened for anthelmintic activity [6,9,10,15]. These assays of adult helminths revealed that antioxidants from both the natural set and synthetic set proved to be effective compounds (Table 1, Figure 2A). Accordingly, in the set of natural compounds, flavonoids turned out to be quite effective anthelmintic agents, in particular, the IC_50_ of quercetin, as the most effective substance, was 5.1 μM. Flavone was also quite effective (IC_50_ = 17.4 μM) against adult liver flukes, as was naringenin (IC_50_ = 14.2 μM). Resveratrol had virtually no anthelmintic activity (IC_50_ of only 259 μM).

Of the synthetic antioxidants, the anthelmintic activity was noted for the sulfur-containing CO3 compound (IC_50_ = 4.6 μm); in addition, oxadiazole was relatively effective (IC_50_ = 21.8 μM). In general, most of the natural and synthetic compounds tested against adult helminths were significantly weaker than PZQ (IC_50_ = 0.47 μM) (Figure 2A).

Nevertheless, one of the antioxidants, in particular, mitochondria-targeted SkQ1, was almost as effective as PZQ (1.4 μM) (Figure 2A). Moreover, SkQ1 caused sloughing of the tegument, which at concentrations of 10 μM was clearly visible as desquamated layers of the tegument (Supplementary Figure S1) at 1 day after the treatment. 

**Figure 3:**
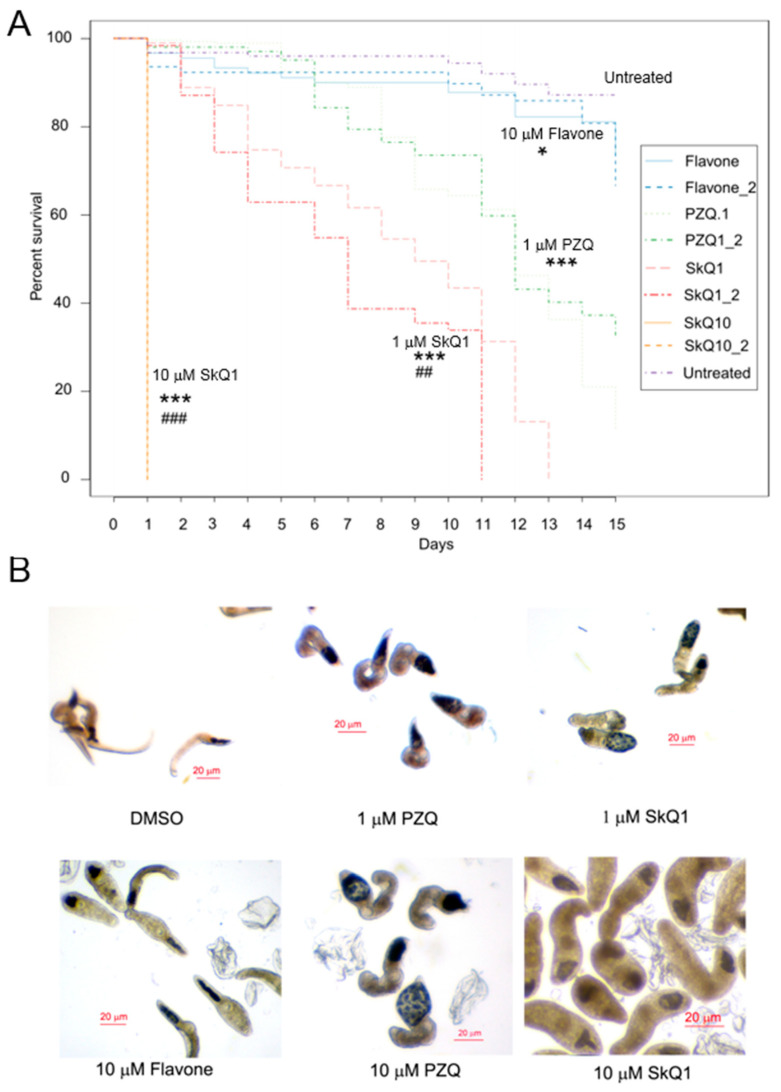
Survival curves and phenotypes of newly excysted metacercariae (NEM) of *Opisthorchis felineus* (*O. felineus)* liver flukes. (**A**) Kaplan–Meier survival curves. Newly excysted metacercariae (NEM) were treated with one of the following compounds: 10 µM flavone, 10 µM 10-(6′-plastoquinonyl)decyltriphenylphosphonium (SkQ1), 1 µM SkQ1, 1 µM praziquantel (PZQ), or dimethylsulfoxide (DMSO). Statistical significance of differences in the survival was evaluated by the log-rank (Mantel–Haenszel) test in pairwise comparisons. The survival curves show statistical significance when 10 µM SkQ1, 1 µM SkQ1, or 1 µM PZQ were compared with the untreated (i.e., DMSO-treated) worms (*p* < 0.0001, ***); when 10 µM SkQ1 (*p* < 0.0001, ^###^) or 1 µM SkQ1 (*p* < 0.01, ^##^) were compared with the 1 µM PZQ group; and when 10 µM flavone was compared with the DMSO group (*p* < 0.05, *) (‘survival’ [v.2.38] R package). (**B**) Phenotypes of NEM of *O. felineus* liver flukes after 1 day of the treatment. Representative photos are shown. The worms were motile and elongated in DMSO group. After 1–10 µM PZQ, and 1 µM SkQ1 treatment, the parasites were contracted and had the swollen, oval-like appearance. High doses of SkQ1 (10 µM) treatment caused darkening of the worms and the development of the characteristic turbidity of the colouring, which means the death of worms.

Motility assays of juvenile helminths, also known as newly excysted metacercariae (NEM) revealed that SkQ1 was also the most active (IC_50_ = 0.6 μM), even better than PZQ (IC_50_ = 0.98 μM) (Figure 2B and Figure 3B). Among the other agents tested on juvenile helminths, flavone was the most active in the set of flavonoids (IC_50_ = 11.6 μM). However, according to phenotypic manifestations, to achieve the same effect as SkQ1’s, a 10-fold higher concentration of flavone was required (Figure 2B and Figure 3B). All other antioxidants showed either weaker or no anthelmintic activity (IC_50_ of 33–1035 μM).

### 2.2. Mortality Assays

A set of Kaplan–Meier survival curves (three independent experiments) is shown in Figure 2A. In the 15-day survival tests, untreated worms (DMSO only) had 88% survival rates (Figure 3A), i.e., the mortality rate was 12% after the 15 days of incubation. PZQ at 1 μM caused the death of 70% of helminths after 15 days of treatment (Mantel–Haenszel test; *p* < 0.001).

We noted a pronounced effect of SkQ1 on the survival rate of helminths. In particular, 10 μM SkQ1 had a 100% lethality rate toward the helminths after 1 day of the treatment (*p* < 0.001). When 1 μM SkQ1 was applied, 100% helminth mortality was recorded after 11–13 days of the treatment, which was significantly higher than that in the 1 μM PZQ group (*p* < 0.01).

Only a small effect on the survival rate of helminths was exerted by 10 μM flavone: mortality by day 15 was 22–35%, which was significantly higher than that of untreated worms (*p* < 0.05). The other antioxidants did not affect the survival rate of helminths at a concentration of 10 μM (data not shown).

The tegument of trematodes is a multinuclear syncytium that is approximately 4 µm thick. The tegument performs several vital functions for the worm, including protection from immune cells of the host, absorption of nutrients, ion transport, and communication with the underlying nervous system [16]. The tegument is in direct contact with muscle fibres, ensuring the instantaneous reaction of the muscles to external stimuli, such as mechanical pressure, or an ion gradient [16]. 

**Figure 4:**
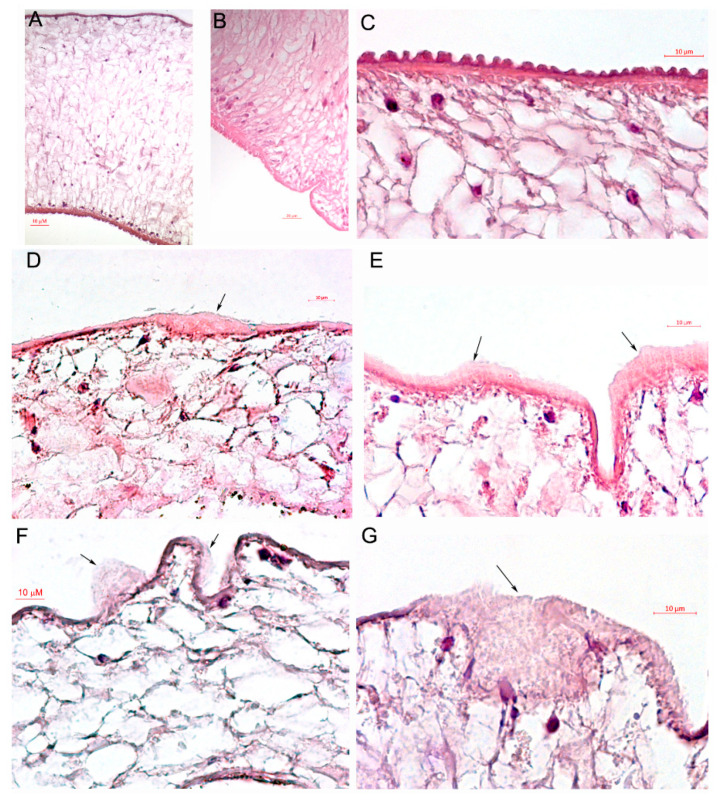
Tegument structure of adult worms at 1 day after the treatment. (**A**–**C**). Adult worm tegument after dimethylsulfoxide (DMSO) treatment. (**D**,**E**) Swelling of the tegument of adult worm, treated with 1 µM praziquantel (PZQ). (**F**,**G**) Swelling and sloughing of the tegument of adult worm, treated with 10 µM 10-(6′-plastoquinonyl)decyltriphenylphosphonium (SkQ1). Five helminths from each group of treatment were analyzed. Representative pictures are shown.

Hematoxylin and eosin (H&E) staining was performed to analyze the tegument structure of adult flukes after SkQ1 treatment. The tegument of adult fluke incubated with DMSO appeared normal (Figure 4A–C). Figure 4D,E depict the swelling of the surface of an adult fluke treated with 1 μM PZQ. Extensive sloughing and swelling of the tegument was detected on the surfaces of most adult flukes treated with 10 μM SkQ1 (Figure 4F,G), which indicated tegumental damage.

## 3. Discussion

In this study, we identified several antioxidants of various chemical classes that may be promising as anthelmintic agents. Promising compounds have been found among both natural and synthetic compounds.

The highest anthelmintic activity among the tested compounds belongs to mitochondria-targeted antioxidant SkQ1. To the best of our knowledge, SkQ1 has never been tested as an anthelmintic agent. In a broad sense, SkQ is a lipophilic cation, linked via a saturated hydrocarbon chain to a natural ubiquinol (coenzyme Q) antioxidant. We demonstrated that the anthelmintic effect of SkQ1 in motility assays was almost the same as that of PZQ, and SkQ1 was significantly more effective than PZQ in mortality assays. Previously obtained results on compounds of ubiquinone-like structure point to the probable mechanism of anthelmintic action of SkQ1. It was reported that the natural trypanocidal ascofurane [7], which has a structure similar to that of ubiquinone, inhibits trypanosome alternative oxidase, in particular, suppresses electron transfer from ubiquinol to oxygen. Trypanosome alternative oxidase is a ubiquinol-dependent terminal oxidase that is located inside mitochondria and is required for aerobic glucose metabolism. This enzyme is present in parasitic organisms only; therefore, it may be a target of ubiquinol-related compounds in liver flukes also and might be investigated as a target for drug development for liver fluke infections.

An oxadiazole derivative (5-phenyl-1,3,4-oxadiazole-2-propionic acid) showed moderate activity against adult helminths in motility assays. The synthesis of oxadiazole derivatives—owing to their broad and promising biological activities—is of interest to medicinal chemists working in drug development. Oxadiazoles manifested high potency with IC_50_ values in a low micromolar range (0.78–22.4 μM) toward *H. contortus* [15], trypanosomes, *Leishmania* [11], and some schistosomes [6]. Oxadiazole activity against *O. felineus* was demonstrated for the first time in our study. 

In the set of sulfur-containing compounds, the anthelmintic activities of СО3 were more pronounced than those of СО4, although the structures of these compounds are very close. СО3 is a multifunctional antioxidant that simultaneously belongs to the classes of sulfur-containing and spatially hindered phenolic organic compounds, is practically nontoxic, and was suggested for the manufacture of food packaging [17].

Traditional medicine in most countries includes natural remedies against parasitic worms, particularly, cattle nematodes [18]. Some phenolic compounds (flavonoids and terpenes) extracted from different, but mostly plant sources, are active against parasitic nematodes [18]. Flavonoids (naringenin, quercetin and luteolin) were shown to be highly effective in vitro at 250 μM concentrations against on *H. contortus* third stage larvae using the larval exsheathment inhibition assay [19]. Luteolin was also found to possess anthelmintic activity against *Trichuris muris* (IC_50_ = 33.9 μM) [20]. Three of the flavonoids (flavone, quercetin, and naringenin) exerted pronounced anthelmintic action in our motility assays. The strongest activities were demonstrated for flavone, which significantly reduced the motility of adult (IC_50_ = 17.4 μM) and juvenile (IC_50_ = 11.4 μM) worms and survival rates of the helminths. 

Another important motivation for using antioxidants as an adjunctive agent in a combination therapy is that in chronic opisthorchiasis, the accumulation of oxidative damage increases with the duration of infection and correlates with the disease severity [3,4]. Moreover, resolution of the pathological hepatobiliary changes is not seen in all cases after the flukes have been eliminated [5,10]. Unfortunately, these individuals may be at high risk of severe hepatobiliary sequelae including cholangiocarcinoma [5]. Antioxidants and hepatoprotective agents are regarded as prime candidates for a combination therapy with PZQ [14]. It is possible that the use of antioxidants can help eliminate excessive accumulation of reactive oxygen and nitrogen species and reduce the risk of cholangiocarcinoma. Antioxidants exert protective effects against liver fibrosis and cholangiocyte hyperplasia during an infection owing to the ability to inhibit host oxidative stress and the formation of toxic products [4] and to affect the mitochondrial activity and fibrotic processes [21,22]. The anti-inflammatory and antifibrotic potential of SkQ1 was demonstrated on other disease models involving chronic inflammation [23].

It is also necessary to discuss the cytotoxicity of the tested drugs. For PZQ, the cytotoxicity data are somewhat inconsistent. According to Sun et al., IC_50_ (PZQ) is 165 µM for human hepatoma HepG2 cells and 102 µM for human fetal hepatocyte L-02 cells at 48 h after treatment [24]. However, according to other authors, only 30% of HepG2 cells remain alive 48 h after treatment with 99 µM PZQ [25]. Unfortunately, in vitro cytotoxicity data against liver cells for SkQ1 are not available. Nazarov et al. tested this compound against Hela cells and found that ED_50_ was about 20 μM [26]. This indicates that SkQ1 might be more toxic than PZQ. Nevertheless, a double-masked clinical study of SkQ1 eye drops in patients showed that it was effective, safe, and well-tolerated for protecting against corneal damage [27]. Although future experiments are needed to study anthelmintic effect of SkQ1 in vivo, our data might help to expand a range of applications of ubiquinone-related compounds and to use the substances in the complex therapy of the liver fluke infections.

## 4. Materials and Methods

### 4.1. Hamsters and Experimental Design

Syrian hamsters (*Mesocricetus auratus*) were purchased from the Animal Facility of the Institute of Cytology and Genetics, Siberian Branch of the Russian Academy of Sciences (ICG SB RAS). All the procedures were in compliance with EU Directive 2010/63/EU for animal experiments. The animals were kept and treated according to the protocols approved by the Committee on the Ethics of Animal Experiments at the ICG SB RAS (Permit Number: 42 of 25.05.2018). Euthanasia was performed via carbon dioxide inhalation, and every effort was made to minimize the suffering of the hamsters.

*O. felineus* metacercariae were collected from naturally infected fish (*Leuciscus idus*) caught in the Ob River near Novosibirsk (Western Siberia) and extracted accordingly [3]. Animals (male Syrian golden hamsters, aged 6 to 8 weeks) were orally infected with 70 *O. felineus* metacercariae.

### 4.2. Drugs

Flavone (2-phenyl-chromone), resveratrol (3,5,4′-trihydroxy-trans-stilbene), PZQ, quercetin, silymarin flavonolignans, and an oxadiazole derivative (5-phenyl-1,3,4-oxadiazole-2-propionic acid) all were purchased from Sigma–Aldrich, St. Louis, MI, USA. SkQ1 (Institute of Mitoengineering of Moscow State University, Moscow, Russia) was kindly provided by Prof. Nataliya Kolosova from ICG SB RAS. CO3 (bis-[3-(3,5-di-tret-butyl-4-hydroxy-phenyl)propyl]-sulfide), CO4 (bis-[3-(3,5-di-tret-butyl-4-hydroxy-phenyl)propyl]-disulfide), and betamide (N-[3-oxo-20(29)lupen-28-oil]-3-aminopropionic acid) were synthesized and provided by Vorozhtsov Institute of Organic Chemistry SB RAS (Novosibirsk, Russia). The chemicals were dissolved in dimethyl sulfoxide (DMSO) (Sigma–Aldrich, USA) to obtain 10 mM stock solutions. 

### 4.3. Motility and Mortality Assays

Newly excysted metacercariae (NEM) were hatched from metacercariae [3,9,28]. Flukes were recovered from the liver of hamsters at 2 months p.i. and thoroughly washed with sterile saline (0.9% NaCl). The worms were incubated at 37 °C for 24 h in the RPMI 1640 culture medium (Life Technologies, Carlsbad, USA) supplemented with 100 U/mL penicillin, 0.1 mg/mL streptomycin, and 0.25 μg/mL amphotericin B (Sigma–Aldrich, St. Louis, USA) and 1% glucose in a CO_2_ incubator [3,9,10].

Worm viability was evaluated under an inverted microscope (Axiovert 40CFL) equipped with a camera (Axiocam ICC3, Zeiss) after 24 h of treatment (magnification 10–50×). The worms were classified as dead if they had a dark color and no movement was observed for 2 min [3,9,28]. For the calculation of half-maximal inhibitory concentration (IC_50_), we tested the following concentrations of compounds: 0.001, 0.01, 0.1, 1, 10, 100, and 500 µM, the dilution factor was 1:200. The solvent concentration across different compound concentrations was 0.5%. Four to five adult worms and 30–40 NEM were used per well. The experiments were repeated three times with two or three biological replicates for each concentration. As control groups, we incubated flukes in the medium with 0.5% DMSO. After 24 h, the motility was evaluated under the light microscope (Axiovert 40CFL; magnification 10–50×) on a motility scale from 0 to 3 (0 = immotile, 1 = very low motility, 2 = low motility, 3 = normal motility) as described previously [3,9,28]. Data obtained from the individual experiments were averaged and adjusted to the data obtained from no-treatment controls. Four-parameter logistic regression was utilized to calculate IC_50_ and standard error (SE) values (‘drc 3.0-1’ R package). The ANOVA lack-of-fit test was performed to assess the hypothesis that a proposed regression model fits the data well. 

For microscopic examination of tegument, adult flukes after the treatment with DMSO, 1 µM PZQ, 10 µM SkQ1 were fixed, processed and stained with hematoxylin and eosin dye as previously described [4].

To estimate the mortality rates, NEM were treated with one of the following agents: 1 µM PZQ, 10 µM SkQ1, 1 µM SkQ1, 10 µM flavone, 10 µM quercetin, 10 µM silymarin, 10 µM resveratrol, or 0.5% DMSO and were incubated at 37 °C for 15 days. The effect of each drug was evaluated three times. To estimate the mortality rates, Kaplan–Meier survival curves were built using the ‘survival’ (v.2.38) R package. Finally, statistical significance of a difference in the survival log-rank (Mantel–Haenszel) test within each pair of samples was calculated. 

## 5. Conclusions

During the past decades, a lot of research has been carried out around antioxidants and their influence on health. It has been emphasized that the balance between oxidation and antioxidation is critical for a healthy biological system. Low doses of antioxidants may be beneficial to this system, but high quantities may disrupt the balance [29]. The limitations of antioxidants and their metabolism still pose a challenge to future research in this field. 

To conclude, SkQ1 and other antioxidant substances appear to have an anthelmintic activity against liver flukes in motility and mortality assays. We propose that structure–activity relationship research might be worthwhile based on the structures of the most effective substances. Studies should also be undertaken to assess possible synergy of combinations of these substances with PZQ against parasites, other trematode species, and liver fluke infections in laboratory animals.

## Figures and Tables

**Table 1:** Motility half-maximal inhibitory concentration (IC_50_) values of anthelminthic activity of antioxidants against newly excysted metacercariae and adult *Opisthorchis felineus* worms.

Compound	Adult Worms		NEM	
	IC_50_ Value, (µM)	SE	IC_50_ Value, (µM)	SE
Praziquantel	0.47	0.05	0.98	0.2
SkQ1	1.4	0.5	0.6	0.2
Quercetin	5.1	0.9	118	70.4
Naringenin	14.2	6.6	80.9	33.8
Flavone	17.4	3.5	11.6	2.7
5-Phenyl-1,3,4-oxadiazole-2-propionic acid	21.8	5.8	1035	272
Sylimarin	56.7	28.3	190	53.4
CO3	4.6	1.3	105.2	21.8
CO4	59.9	26.9	33.1	11.4
Betamide	167	27	261	36
Resveratrol	259	26	80.9	13.6

Data are presented as IC_50_ values, SE: standard error (‘drc 3.0-1’ R package).

## Data Availability

All data generated or analyzed during this study are included in this published article.

## Supplementary Materials

The following are available online at https://www.mdpi.com/2076-0817/10/3/284/s1, Supplementary Figure S1: The phenotypes of adult *O. felineus* liver flukes after 1 day of the treatment in vitro.
