# Supplementary material for: Anthelmintic Activity of Antioxidants: In Vitro Effects on the Liver Fluke Opisthorchis felineus
Source: Pathogens. 2021 Mar 2;10(3):284. doi: 10.3390/pathogens10030284 (PMC8001094; doi:10.3390/pathogens10030284)
Supplement: Supplementary file 1 [file pathogens-10-00284-s001.pdf]

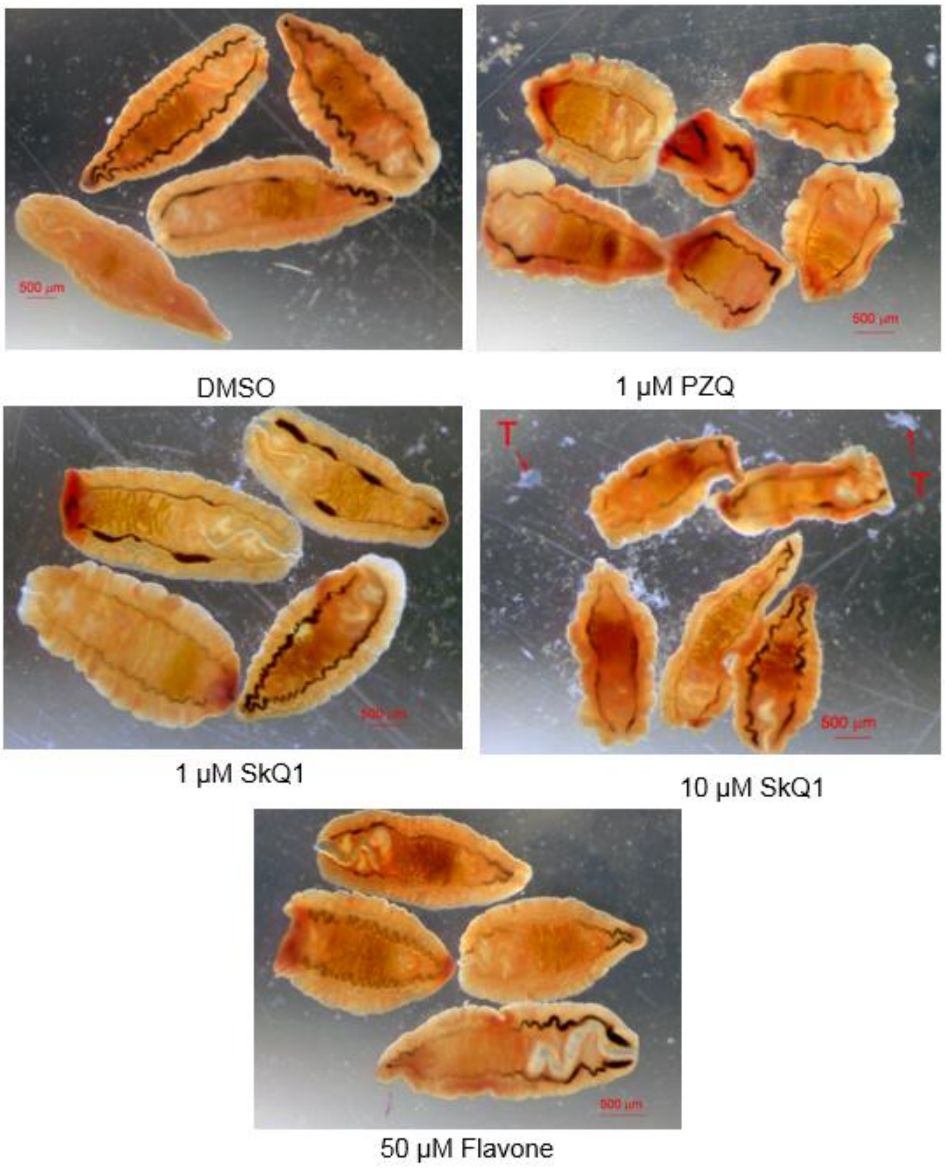

Supplementary Figure S1. The phenotypes of adult *O. felineus* liver flukes after 1 day of the treatment *in vitro*. Pieces of the helminth shell, exfoliated from the surface of the helminth after the 10 µM SkQ1 treatment, are pointed out by arrows and the letter T (tegument).
